# Duckweed: Research Meets Applications

**DOI:** 10.3390/plants12183307

**Published:** 2023-09-19

**Authors:** Viktor Oláh, Klaus-Juergen Appenroth, K. Sowjanya Sree

**Affiliations:** 1 Department of Botany, Institute of Biology and Ecology, Faculty of Science and Technology, University of Debrecen, 4032 Debrecen, Hungary; 2 Matthias Schleiden Institute–Plant Physiology, Friedrich Schiller University Jena, 07743 Jena, Germany; 3 Department of Environmental Science, Central University of Kerala, Periye 671320, India

## 1. Introduction

**Figure 1:**
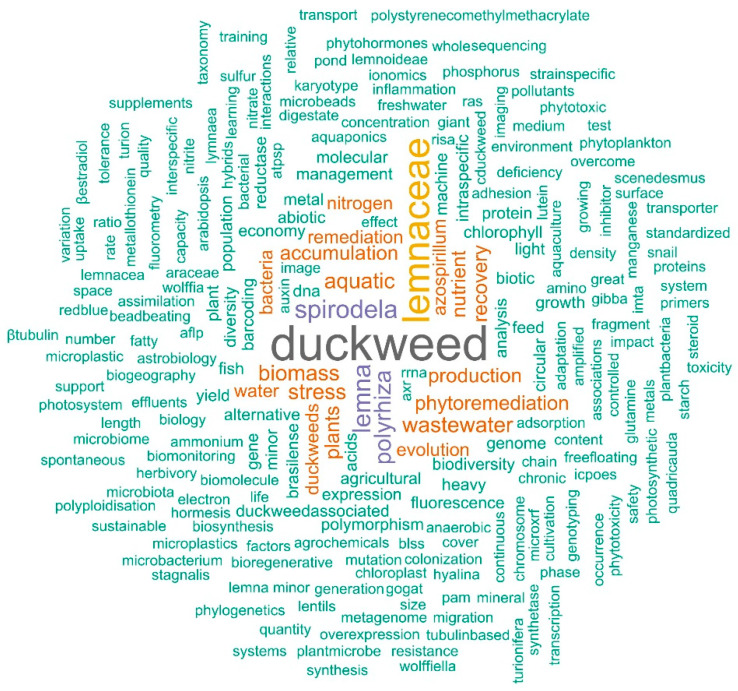
A word cloud of the keywords used in the studies published in the Special Issue “Duckweed: Research meets applications”. The font size of the words indicates their occurrence frequency throughout the Special Issue.

**Figure 2:**
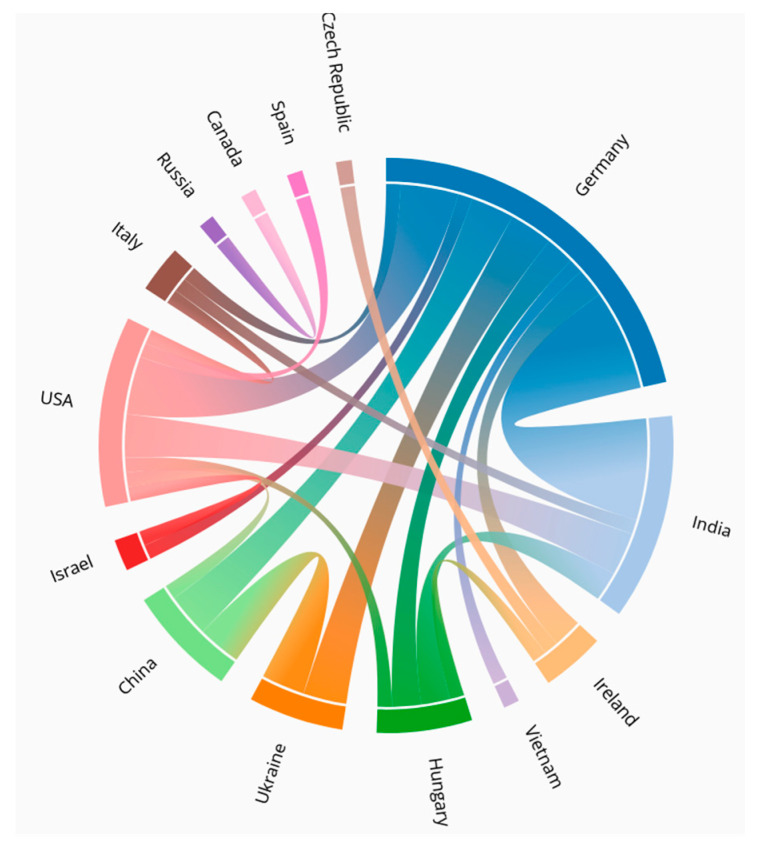
International linkages between different countries cooperating on diverse fields of duckweed research and applications, based on the publications in this Special Issue.

The Special Issue “Duckweed: Research Meets Applications” of the journal *Plants* (ISSN 2223-7747) presents a comprehensive update of the current progress in the field. It includes a total of 38 articles, 29 original research papers, 5 reviews, 2 conference reports and 2 communications, encompassing almost all areas of research and applications related to the aquatic monocotyledonous plants duckweeds. The content of this Special Issue reflects the diversity of the duckweed community well in terms of the focal areas of research (Figure 1) as well as the international linkages (Figure 2). The authors are affiliated to a total of 18 countries: Belgium, Canada, China, Czechia, Germany, Hungary, India, Ireland, Israel, Italy, Poland, Russia, Slovenia, Spain, Thailand, Ukraine, the USA and Vietnam (in alphabetic order), and exactly half of the papers (19 out of 38) were an outcome of international collaborations. The original deadline of article submission to this Special Issue was extended in order to provide an opportunity to the participants of the 6th International Conference on Duckweed Research and Applications (ICDRA), which was held in Gatersleben, Germany, from 29 May to 1 June 2022. A report on this conference, “Sixth International Conference on Duckweed Research and Applications Presents Lemnaceae as a Model Plant System in the Genomics and Postgenomics Era” [1], presents the enormous progress made in duckweed research and applications since the first ICDRA in 2011 [2]. Interestingly, also in 2022, a workshop was held at the University of Jena, comparing the stress responses of duckweeds (aquatic plants) and terrestrial plants, a report of which is presented in this Special Issue as well [3]. In a similar direction, Ziegler et al. [4] reviewed the present knowledge of “Survival Strategies of Duckweeds”. The survival strategies in duckweeds represent the natural potential of these plants to withstand several unfavourable conditions.

Together with a recently published review article [5], the articles in this Special Issue offer a fast overview of the present state of the art in duckweed research. So far, the papers published in this Special Issue have been accessed >75,000 times through the journal’s homepage alone and received 161 citations in total as of 4 August 2023.

## 2. Molecular Characterization and Taxonomy

The family Lemnaceae was circumscribed by Ivan Martinov (1771–1833) as early as 1820. Therefore, the valid name of the family is Lemnaceae Martinov [6]. With the beginning of the era of molecular taxonomy, the closeness of this family to Araceae became evident [7] and the Angiosperm Phylogeny Group (APG) integrated this plant family as the subfamily Lemnoideae into the expanded family Araceae. Tippery et al. [8] demonstrated that this is not a vital step following the taxonomic rules. They suggested instead to restore the Araceae subfamily Orontioideae as the family Orontiaceae, which makes it possible to keep the family rank of the long-recognized family Lemnaceae. This results in three distinct lineages as families: Araceae s.s., Lemnaceae and Orontiaceae [8]. These authors also showed that the change of family Lemnaceae into a subfamily of Araceae was not well accepted by the scientific community, and this holds true for all papers published in this Special Issue. More than 10 years after the suggestion to treat duckweeds as an Araceae subfamily, it might be time to revise this on the basis of de facto use and as further advocated for by Tippery et al. [8]. A landmark in duckweed taxonomy was presented by the group of Laura Morello [9], describing the discovery of interspecific hybrids in the *Lemna* genus (*L. minor*, *L. gibba*, *L. turionifera*) using the method of tubulin-gene-based polymorphism (TBP; cf. also [10]). This provides a new insight into the evolution of duckweeds. Besides the identification of duckweed species, the identification of clones of the same species is also important, especially for several practical applications, including patenting. Bog et al. [11] used five orthogonal molecular methods, NB-ARC-related genes, TBP, simple sequence repeats (SSRs), multiplexed inter-simple sequence repeat genotyping by sequencing (MIG-seq) and genotyping by sequencing (GBS), for this purpose. Whereas TBP could distinguish only 7 clones out of 23 of *Spirodela polyrhiza*, the other four methods could distinguish 20 to 22 genotypes. *Spirodela polyrhiza* was selected for these test methods because it is known that it has especially low intraspecific variation [12]. This also became evident in a project where samples of *S. polyrhiza* and *L. minor*, collected around small ponds, were investigated using amplified fragment length polymorphism (AFLP). Whereas several distinct clones were identified within the populations of each pond in the case of *L. minor*, *S. polyrhiza* clones only showed genetic differences between the ponds [13]. Using plastid barcoding (*atp*F-*atp*H and *psb*K-*psb*I), Chen et al. [14] identified six different species from Ukraine and six different species from Eastern China. Interestingly, *Lemna aequinoctialis* did not form a uniform taxon, which might be a hint for the existence of hybrids. The same plastidic markers for barcoding were used by Yosef et al. [15], identifying six different species from Israel. For several investigations, the knowledge of the number of chromosomes in the different duckweed species and clones is important, and Hoang et al. [16] summarized it for all 36 duckweed species. However, only half of them were investigated by this group, whereas the other half were taken from results published mainly by Urbanska [17] and Geber [18]. These two authors reported unusually high variations in chromosome numbers within the same species, which need to be reviewed with care. A spontaneous mutant of *L. gibba* G3 was shown to be tetraploid [19]. This mutant is significantly larger in several parameters at morphological and anatomical levels. Also, at the physiological level, differences were found in the flower induction patterns, wherein the tetraploid plants flowered under conditions that were non-inductive for the diploid plants. Moreover, the transcript levels of nuclear genes of the photosynthetic apparatus were expressed at a higher level in the tetraploid plants when compared to the diploid ones.

## 3. Phytoremediation: Wastewater

Water pollution and meeting the ever-increasing clean water demands are interconnected problems that our modern society must tackle. Duckweeds have long been considered as potent candidates for wastewater management. As reviewed by Zhou et al. [20], these fragile plants were true giants in reclaiming contaminated waters while providing valuable biomass at the end of the process.

Six case studies examined the performance and pitfalls of duckweed-based wastewater remediation systems. Using multi-tiered indoor bioreactors, Coughlan et al. [21] studied how to design the water depth and flow rate of such systems to minimize the physical disturbance of plants while maintaining efficient mixture of the medium. Devlamynck et al. [22] and Lambert et al. [23] studied flow-through and recirculating systems for treating swine manure effluents with duckweeds and discussed the risks and possible solutions for avoiding or managing the accumulation of various chemical elements. Walsh et al. [24] and O’Mahoney et al. [25] focused on the possible valorisation of waste streams from dairy processing by studying whether this kind of medium could support the cultivation of duckweeds and how plant density affected the nutrient removal efficiency of the system. Paolacci et al. [26] addressed another applied field by analysing the actual contribution of duckweeds, phytoplankton and biofilm to the nutrient-removing performance of a duckweed-based aquaculture wastewater remediation system.

A series of studies addressed basic physiological processes that make duckweeds efficient in water remediation and waste valorisation. Zhou et al. [27], by studying species from four genera, analysed whether the preference for either NO_3_^−^ or NH_4_^+^ as inorganic nitrogen sources was general or rather species-specific amongst Lemnaceae. In addition, they provided insights to the complex regulation of nitrogen assimilation in these plants by reporting the molecular structure and differential expression of several key enzymes in response to different inorganic nitrogen sources. Nitrogen availability is not only crucial in the plants’ nutritional status, but may also modulate responses to other stressors, such as the presence of heavy metals. In their study, Kishchenko et al. [28] focused on the mechanisms by which NH_4_^+^ could mitigate manganese toxicity in *S. polyrhiza*, including the interactions between Mn availability and the transcription of ammonium transporters. Besides remediation, metal accumulation may also gain significance when future duckweed-based feed and food production is considered. Accordingly, Pakdee et al. [29] identified metallothionein-like genes and analysed dynamics in their transcript abundances under Cu and Cd stress, while, in another study, Oláh et al. [30] compared changes in the biomass ionomic composition of different duckweed species in response to acute Ni and Cr(VI) stress and the localization of heavy metal accumulation in the fronds.

## 4. Applications: Accumulation of Protein or Starch

Application of duckweed on an industrial or commercial scale requires the production of large amounts of biomass. This can be attained either by using very large water surface areas or by using a large number of smaller facilities in a modular arrangement. Wastewater cleaning with typically very high volumes of liquid waste might be preferentially carried out outdoors in large ponds or waterways [31]—although indoor treatment might be possible. Modular arrangement might be especially useful when specific environmental conditions should be applied in order to produce biomass with a specific quality. This is the domain of indoor cultivation. Petersen et al. [32] investigated the growth rates and protein contents of *L. minor* and *Wolffiella hyalina* in an indoor experiment under the influence of different nitrate-to-ammonium ratios. The best results were obtained in a 50% diluted N medium with a nitrate-N to ammonium-N ratio of 3:1. In an additional set of experiments, the influence of light intensity and light source spectrum was investigated in a “small-scale, re-circulating indoor vertical farm” [33] providing the pre-condition for further upscaling of the platform (cf. [34]). The same group used the produced biomass successfully for feeding broiler chickens [35]. Romano et al. [36] discussed a somewhat uncommon realm of indoor cultivation in their review paper, that is, the potentials of *Wolffia* species in space applications. As the authors pointed out, the world’s smallest plants are promising candidates to be incorporated into bioregenerative life support systems in long-term space missions, and they are able to recycle water and oxygen for astronauts while also providing them fresh vegetable biomass.

A group from Italy substituted different amounts of the standard feed of rainbow trout on the protein basis and reported that 20% substitution did not have any negative effects on the fish, but 28% substitution did show effects such as reduced fish body weight and a few other parameters [37]. Demmig-Adams et al. [38] stressed that duckweeds (*Lemna*) have very high growth rates combined with unusually high levels of zeaxanthin, which is important for human nutrition. Moreover, *Lemna* plants can respond to elevated CO_2_ concentrations with increasing growth rates. It has been known for a long time that under stress conditions, the protein content of duckweeds decreases as strongly as the starch content increases. The accumulation of starch in a large number of duckweed species under nutrient-limited cultivation conditions (phosphate, nitrate, sulphate) has been shown [39]. Starch contents of 50% per dry weight can be reached, making the biomass suitable for energy production, e.g., through saccharification and fermentation to bioalcohols. As a link between carbon, nitrogen and sulphur homeostasis, overexpression of the phosphoserine phosphatase-encoding gene was found to promote starch accumulation in *Lemna turionifera* under sulphur limitation in the study of Wang et al. [40].

## 5. Interaction with Microorganisms

The interaction of duckweeds with microorganisms, especially plant-growth-promoting bacteria, can result in enhanced plant growth [41]. The identification of bacteria associated with the root–frond interface on several duckweed species is important in this field. Acosta et al. [42] described the development of a generic fingerprinting assay that included genomics-based bacterial-strain-specific primers, making it possible to distinguish strains from the same genus. Quantification was possible by using plant reference genes. Many of these bacterial strains produced indole-related compounds like auxins. Gilbert et al. [43] identified suitable bacterial strains and investigated morphological responses of duckweeds as consequences of this interaction. Communities of duckweed-associated bacteria depend on external factors, e.g., those connected with stress induced by nutrient deficiency. Bunyoo et al. [44] were able to show that the relative abundance of bacteria, e.g., from the genus *Rhodobacter*, changes after transfer from conditions in nature to nutrient-deficient conditions. Some herbivores feed on duckweeds, e.g., on *S. polyrhiza*. Schaefer and Xu [45] quantified the influence of duckweed-associated bacteria on the fitness of plants evaluated using the extent of herbivory by the great pond snail. The six genotypes of *S. polyrhiza* tested did not differ in their resistance toward the herbivore. However, after outdoor inoculation with microbiota associated with the same plant species, altered herbivory tolerance was observed in a genotype-specific manner.

## 6. Physiology and Phytomonitoring

Duckweeds are ideal for studying hormonal effects in plants, as they can be cultured axenically and take up substances directly into the shoot. As a vivid example of this traditional role, Chmur and Bajguz [46] analysed growth responses to brassinosteroids in parallel with other biochemical parameters in *Wolffia arrhiza* treated with brassinolide and brassinazole, a synthetic brassinosteroid inhibitor. Kozlova and Levin [47], on the other hand, studied plant responses by *L. minor* to a fish steroid, 17β-Estradiol, that has been released at a large scale by intensive fish farms, and they found growth-promoting effects.

Besides plant physiology research, bioindication of pollutants is another classical field of duckweed applications. Microplastics are emerging contaminants and, as such, their potential risks need to be explored urgently. Two studies aimed to fill this knowledge gap: Rozman and Kalčíková [48] tested if duckweeds could be used in monitoring microplastic contamination of freshwaters by adsorbing these particles on their surface. Ceschin et al. [49] focused on the morphological and biochemical responses of these plants to microplastics, comparing acute effects to the chronic ones over an extended exposure period. Two further studies addressed methodological aspects of working with duckweeds: Oláh et al. [50] reviewed the vast diversity of various chlorophyll fluorescence-derived phytotoxicity endpoints that were reported in the literature on the application of the chlorophyll fluorescence imaging method on duckweeds and compared the responsivity of some widely used parameters to different toxicants. As another approach, Romano et al. [51] developed a workflow for automated frond surface area quantification by using digital images and machine learning. This method promises to significantly reduce the need for human input in applications that rely on duckweed growth.

## 7. Future Outlook

The present Special Issue gives an update on the state of the art of duckweed research and applications and, together with the two previous Special Issues on this topic [52,53], demonstrates the enormous progress made in this field during the last decade. Nevertheless, there are serious challenges both in research and applications. Duckweeds propagate dominantly by vegetative means but, being angiosperms, they are capable of flowering and seed production, which has been shown in several species. Artificial cross-fertilization and production of seeds has been demonstrated as a viable technique [54]; however, it is not yet a routine procedure. Additionally, genetic modification has been demonstrated by several research groups using various duckweed species but is also not yet a routine method. In most cases, the efficiency is still too low and needs more research. A bottleneck for the transition from research to applications is still the production of large amounts of biomass for any kind of industrial use, be it for food, feed or as energy source. Important steps were already made by the contributions of Petersen et al. [33,34] and by the group of Marcel Jansen (e.g., [25]). Several steps ahead in research and applications were mentioned in the conference report of the 6th ICDRA in Gatersleben, Germany, in 2022 [1]. The 7th International Duckweed Conference will be held in November 2024 in Thailand and we can expect interesting new results in the field.
